# Lightweight Feature Enhancement Network for Single-Shot Object Detection

**DOI:** 10.3390/s21041066

**Published:** 2021-02-04

**Authors:** Peng Jia, Fuxiang Liu

**Affiliations:** Key Laboratory of Dynamics and Control of Flight Vehicle, Ministry of Education, Beijing Institute of Technology, Beijing 100081, China; jiapeng@bit.edu.cn

**Keywords:** object detection, real-time, adaptive receptive field fusion, enhanced up-sampling

## Abstract

At present, the one-stage detector based on the lightweight model can achieve real-time speed, but the detection performance is challenging. To enhance the discriminability and robustness of the model extraction features and improve the detector’s detection performance for small objects, we propose two modules in this work. First, we propose a receptive field enhancement method, referred to as adaptive receptive field fusion (ARFF). It enhances the model’s feature representation ability by adaptively learning the fusion weights of different receptive field branches in the receptive field module. Then, we propose an enhanced up-sampling (EU) module to reduce the information loss caused by up-sampling on the feature map. Finally, we assemble ARFF and EU modules on top of YOLO v3 to build a real-time, high-precision and lightweight object detection system referred to as the ARFF-EU network. We achieve a state-of-the-art speed and accuracy trade-off on both the Pascal VOC and MS COCO data sets, reporting 83.6% AP at 37.5 FPS and 42.5% AP at 33.7 FPS, respectively. The experimental results show that our proposed ARFF and EU modules improve the detection performance of the ARFF-EU network and achieve the development of advanced, very deep detectors while maintaining real-time speed.

## 1. Introduction

Object detection is the most fundamental task in the computer vision community and has attracted researchers’ attention in different fields. Object detection is not only widely used in video surveillance [1] and self-driving [2] but also forms a key component of many other visual tasks, such as scene understanding [3,4] and image guidance [5]. In recent years, the rapid development of deep Convolutional Neural Networks (CNNs) and well-annotated datasets [6,7] has resulted in many breakthroughs by the computer community. Some studies [8,9,10] have enhanced the ability of the model to perform feature extraction, selection and fusion. However, it is still a challenge to carry out real-time and high-precision detection for objects at different scales. At present, advanced detectors improve the performance of object detection by constructing deep feature pyramids and spatially receptive field modules.

To make the detection results more reliable, researchers use a deep feature pyramid network [11,12,13] to obtain multi-scale feature representation in training and testing and improve the detection performance of objects with different scales. SSD [14] is the first attempt to apply the convolution pyramid feature representation to object detection. Given an input image, SSD calculates multi-scale feature maps through forwarding propagation and then reuses multi-scale feature maps to predict different scales of objects. However, the bottom-up path in SSD causes the shallow layer of the model to lack semantic information, making the detection of small instances a bottleneck. To solve the shortcomings of SSD, some works (FPN [11], DSSD [15]) have combined low-resolution, strong semantic features with high-resolution, weak semantic features to build a feature pyramid with a top-down path. By sharing the rich semantic and fine-grained information in the feature pyramid, the model’s detection performance of the object is improved. However, when constructing the feature pyramid, FPN and other top-down structures use the nearest neighbor interpolation method for up-sampling, which takes the nearest neighbor point’s gray value as the gray value of the point, regardless of the influence of other adjacent pixels. The feature map generated by this method will appear obviously jagged in the place where the gray value changes, causing information loss. To solve this problem, we propose an enhanced up-sampling (EU) module that uses dilated convolutions [16] with different dilation rates for the convolution of the up-sampled feature map, making full use of the context information around pixels to reduce the information loss. By using the EU module, the shallow semantic information in the feature pyramid is enhanced, and the performance of small object detection is improved.

In recent years, to enhance the lightweight model’s feature representation ability and improve the detector’s performance, many studies have explored the spatial receptive field (RF) [17,18] of a CNN. The Inception method [19] uses convolution kernels of different sizes to build a multi-branch structure to diversify the RF. However, as the convolution kernel size increases, the computational cost increases significantly, which reduces the efficiency of the model. The deformable convolutional network (Deformable CNN) [20] learns the offset to adaptively adjust the spatial distribution of RF according to the object’s scale and shape. Although its grid sampling method is very flexible, it does not consider the effect of the RF’s eccentricity on the feature map, which leads to the equal contribution of all pixels in the RF to the output response. Besides this, the receptive field block (RFB) [21] attempts to simulate different RFs in the human visual system through a multi-branch structure and dilated convolution. However, the feature maps generated by different RF branches are simply concatenated in the channel dimension. The contribution of each feature map is treated equally, which reduces the amount of useful information. In this study, to make full use of the feature information of different RF branches and enhance the feature representation ability of the RF module, we propose an adaptive receptive field fusion (ARFF) module. The ARFF module adaptively learns the fusion weights of different RF branches in the RF module, enhances the model’s feature representation ability and improves the detector’s performance.

Some studies [22,23,24] have confirmed that deep backbones can enhance the model’s feature representation ability and improve the detector’s performance. However, the deep network will greatly increase the model’s computational cost and reduce the inference speed. To solve the above problems, we propose a one-stage, lightweight model to improve the detector’s performance while maintaining real-time speed. In Section 3.3, we build a lightweight object detector based on YOLO v3 [25]. We embed the ARFF module into the prediction layer of the feature pyramid to enhance the model’s feature representation ability and improve the detector’s performance. We embed the EU module into the lateral connection of the feature pyramid to enhance the semantic information of the shallow feature maps and improve the detection performance of the detector for small objects. By embedding ARFF and EU modules on top of YOLO v3, we build a lightweight object detector, referred to as the ARFF-EU network. This enhances the discrimination and robustness of model extraction features and achieves the development of advanced, very deep detectors while maintaining the real-time speed of object detection.Our main contributions can be summarized as follows:

- We propose an ARFF module that adaptively learns the fusion weights of different RF branches in the RF module to enhance the model’s feature representation ability.
- We propose an EU module that can reduce the information loss in the up-sampling process, enhance the semantic information in the shallow layer of the feature pyramid and improve the detection performance of small objects.
- We integrate the ARFF and EU modules on top of YOLO v3 to build a lightweight object detector that achieves the development of advanced, very deep detectors while maintaining real-time speed.

## 2. Related Works

### 2.1. Object Detection

With the development of deep learning, the performance of object detectors based on CNN has been significantly improved. The feature pyramid representation is the basis of the multi-scale processing of object detectors. To improve the detection performance of objects at different scales, in [22,26,27], the authors build an image pyramid by inputting multi-resolution images into the training and testing network to obtain multi-scale representations. However, the image pyramid method is time-consuming, and it is difficult to achieve real-time speed. In [9], the authors combine the multi-layer features before making predictions to improve the performance of the detector. SSD attempts to apply convolution pyramid feature representation to object detection using multi-scale prediction layers to detect objects of different sizes. Recently, to make full use of the deep semantic information and shallow fine-grained information in the network, lateral connection [28] has become increasingly popular and has been used in object detection [11,15]. FPN and other top-down structures are simple and efficient, but there is a loss of information in the lateral connection of the feature pyramid. We propose the EU module to reduce the information loss caused by up-sampling in the lateral connection and enhance the representation ability of the feature pyramid. The EU module makes full use of the context information around the pixels to enhance the shallow semantic information in the feature pyramid and improve the detection performance of small objects.

### 2.2. Receptive Field

Increasing numbers of researchers are studying the RF of detectors with the CNN to improve the performance of lightweight detectors. The Inception method uses convolutional kernels of different sizes to build multi-branching structures to diversify RF and capture multi-scale information. Its variants have achieved competitive results in object detection and classification tasks. However, in order to obtain rich context information, a larger convolution kernel is needed, which significantly increases the computational cost. ASPP [29] expands the RF of the deep network without increasing the amount of calculation by the convolution kernel, captures multi-scale context information and improves the model’s feature representation ability. The deformable CNN learns the offset to adaptively adjust the RF’s spatial distribution according to the scale and shape of an object. Although its grid sampling method is flexible, it does not consider the effect of the RF’s eccentricity on the feature map, resulting in the equal contribution of all pixels in the RF to the output response. The RFB network attempts to simulate the RFs of different sizes in the human visual system through a multi-branch structure and dilated convolution and concatenates the feature maps in the channel dimension. However, the feature maps of different RF branches in the RFB network are treated equally, which reduces the amount of useful information. To solve the above problems, we propose the ARFF module in this study, which adaptively learns the fusion weights of different RF branches to enhance the model’s feature representation ability.

## 3. Methods

In this section, we will introduce the ARFF and EU modules, respectively, and describe the architecture of the ARFF-EU network.

### 3.1. Adaptive Receptive Field Fusion

**Figure 1:**
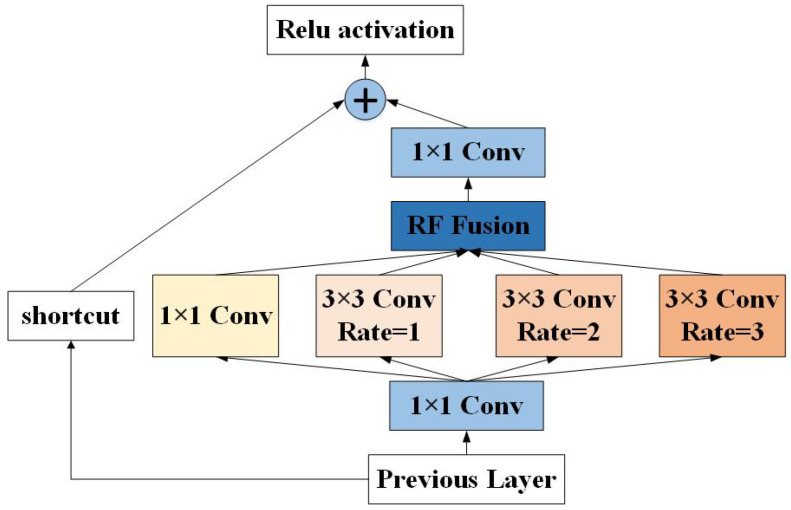
The architecture of adaptive receptive field fusion (ARFF).The rate represents the dilation rate of the dilated convolution. The receptive field (RF) fusion block represents the receptive field fusion module, which is illustrated in Figure 2.

To make full use of the feature maps of different RF branches in the RF module, filter out redundant information, combine useful information and improve the discriminability and robustness of the features extracted by the model, we propose the ARFF module. The ARFF module uses the multi-branch RF structure to generate feature maps with different RFs and adaptively learns the feature maps’ fusion weight through the network to improve the model’s feature representation ability. As shown in Figure 1, the internal structure of the ARFF module can be divided into three main parts: a bottleneck structure, multi-branch receptive field structure and receptive field fusion module. We describe these three parts and their functions in detail below.

Bottleneck structure: As shown in Figure 1, the ARFF module uses a 1 × 1 convolution layer to construct the bottleneck structure. By using 1 × 1 convolution, the number of channels of the previous layer is reduced to one-fourth of the original, which reduces the number of parameters and makes the training more efficient. Finally, we use 1 × 1 convolution to adjust the feature map’s number of channels to enhance the model’s feature representation ability.

Multi-branch receptive field structure: Inspired by RFB Net, we use four convolution branches with different RFs to simulate the RF in the human visual system in this study, and use Ln(n ϵ 0,1,2,3) to represent these branches, as shown in Figure 1. We use a 1 × 1 convolution and three 3 × 3 dilated convolutions to simulate RFs of different sizes, making full use of the feature map’s context information to enhance the feature representation ability of the model.

**Figure 2:**
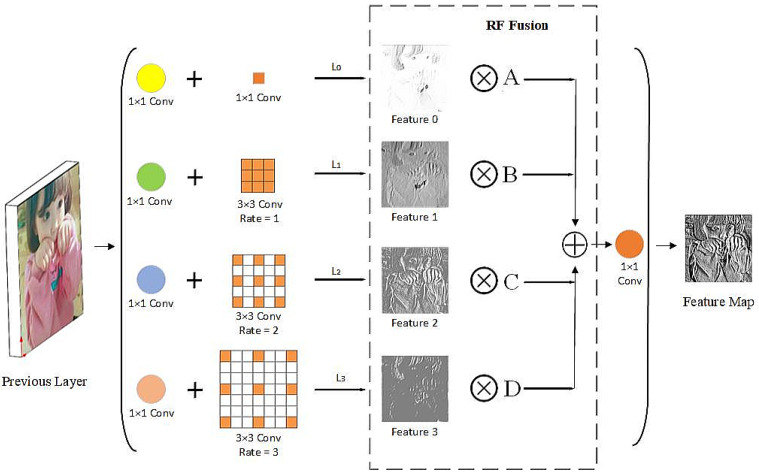
Illustration of RF fusion module. Features 0, 1, 2 and 3 represent feature maps with different RFs. A, B, C and D represent the fusion weight of the feature map. ⨂ represents element-wise multiplication. ⨁ represents the weighted fusion of the feature map.

Receptive field fusion module: As shown in Figure 2, we propose an RF fusion module to fully use the output feature maps in the multi-branch RF structure. This module improves the feature representation ability of the RF module by filtering out redundant information in the feature maps and combining useful information. In this study, $X^{n}$ represents the feature map output from the Ln branch in the multi-branch RF structure and $X_{ij}^{n}$ represents the vector of the feature map $X^{n}$ at position (i,j ). To make full use of the useful information in the branches of different RFs and filter out the redundant information, we propose the fusion of the feature maps at the corresponding position as follows:

(1) $$Y_{ij}=X_{ij}^{0}\times A_{ij}+X_{ij}^{1}\times B_{ij}+X_{ij}^{2}\times C_{ij}+X_{ij}^{3}\times D_{ij}$$

where $A_{ij}$, $B_{ij}$, $C_{ij}$ and $D_{ij}$ represents the fusion weight of the feature map $X^{n}$, which is obtained by the adaptive learning of the network and shares the weight in all channels. $Y_{ij}$ represents the vector of the output feature map in the position (i,j). Inspired by [30,31], we define $A_{ij}$, $B_{ij}$, $C_{ij}$, and $D_{ij}$ as follows:

(2) $$\begin{array}{cc} \; & A_{ij}+B_{ij}+C_{ij}+D_{ij}=1 \end{array}$$

(3) $$\begin{array}{cc} \; & A_{ij},B_{ij},C_{ij},D_{ij}\epsilon \left[0,1\right] \end{array}$$

(4) $$\begin{array}{cc} \; & A_{ij}=\frac{e^{\theta_{A_{ij}}}}{e^{\theta_{A_{ij}}}+e^{\theta_{B_{ij}}}+e^{\theta_{C_{ij}}}+e^{\theta_{D_{ij}}}} \end{array}$$

(5) $$\begin{array}{cc} \; & B_{ij}=\frac{e^{\theta_{B_{ij}}}}{e^{\theta_{A_{ij}}}+e^{\theta_{B_{ij}}}+e^{\theta_{C_{ij}}}+e^{\theta_{D_{ij}}}} \end{array}$$

(6) $$\begin{array}{cc} \; & C_{ij}=\frac{e^{\theta_{C_{ij}}}}{e^{\theta_{A_{ij}}}+e^{\theta_{B_{ij}}}+e^{\theta_{C_{ij}}}+e^{\theta_{D_{ij}}}} \end{array}$$

(7) $$\begin{array}{cc} \; & D_{ij}=\frac{e^{\theta_{D_{ij}}}}{e^{\theta_{A_{ij}}}+e^{\theta_{B_{ij}}}+e^{\theta_{C_{ij}}}+e^{\theta_{D_{ij}}}} \end{array}$$

where $A_{ij}$, $B_{ij}$, $C_{ij}$ and $D_{ij}$ are defined by using the softmax function with $\theta_{A_{ij}}$, $\theta_{B_{ij}}$, $\theta_{C_{ij}}$, and $\theta_{D_{ij}}$ as control parameters, respectively. We use 1 × 1 convolution to adjust the number of channels of the feature map $X^{n}$ to four to calculate the weighted scalar maps $\theta_{A}$, $\theta_{B}$, $\theta_{C}$ and $\theta_{D}$, and they can be learned by standard back-propagation.

By adaptively learning the fusion weights of different RF branches in the multi-branch RF structure, the ARFF module enhances the discrimination and robustness of the features extracted by the model and improves the detector’s performance.

### 3.2. Enhanced Up-Sampling

Recently, object detection algorithms have used lateral connections to build feature pyramids, enhance the semantic information of shallow feature maps and improve the detection performance of small objects. It is simple and efficient to use the nearest neighbor interpolation method for up-sampling in the lateral connection, which takes the nearest neighbor point’s gray value as the gray value of the point, regardless of the influence of other adjacent pixels. However, the gray value of the feature map generated by this method is discontinuous, and an obvious jagged shape may appear in the place where the gray level changes, resulting in the loss of semantic information. To eliminate the information loss caused by up-sampling, enhance the semantic information of the shallow feature maps in the feature pyramid and improve small object detection performance, we propose the EU module in this study.

**Figure 3:**
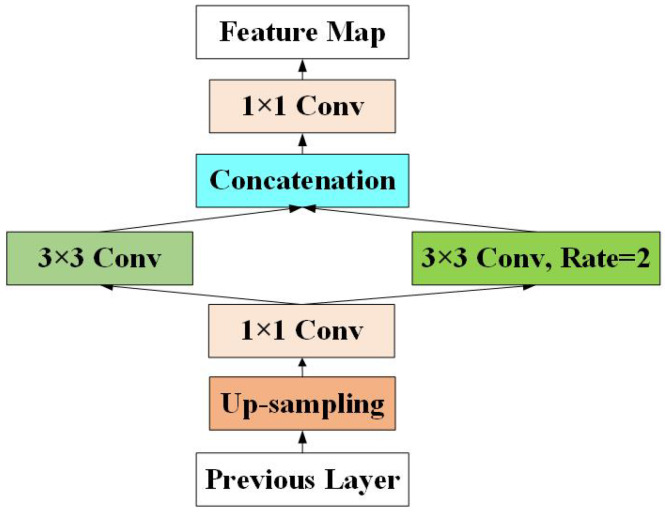
The architecture of the enhanced up-sampling (EU) module.

As shown in Figure 3, the internal structure of the EU module can be divided into four main parts: an up-sampling layer, bottleneck structure, feature enhancement layer and concatenation layer. First, we use the up-sampling layer to adjust the feature map’s size in the lateral connection. Then, we use 1 × 1 convolution to adjust the number of channels of the up-sampling output feature map to the original one or two, which reduces the number of parameters and improves the model’s training and testing efficiency. To make full use of the context information around pixels and eliminate the information loss caused by up-sampling, we use 3 × 3 dilated convolution with two different dilation rates to construct the feature enhancement structure. Finally, the concatenation layer connects the feature map output by the feature enhancement layer in the channel dimension and uses a 1 × 1 convolution layer to merge them fully.

The EU module reduces the information loss caused by up-sampling, enhances the semantic information of the shallow feature maps in the feature pyramid and improves the detection performance of small objects.

### 3.3. ARFF-EU Network Detection Architecture

In this study, we built our detection architecture based on YOLO v3. As is well known, the YOLO v3 framework is simple and efficient and mainly consists of two modules: an efficient backbone (Darknet-53) and a feature pyramid network with three levels. Although many accomplished lightweight networks (VGG [14], MobileNet [32] and ShuffleNet [33]) have been proposed recently, in order to achieve a direct comparison with the original YOLO v3, we use Darknet53 as the backbone in this study. To better demonstrate the effectiveness of ARFF and EU modules, we introduced the guided anchor [34] method and the “bag of freebies [35]” tricks into the YOLO v3 framework, almost all of which are free to use, to build a more powerful baseline network, called the baseline. As shown in Table 1, Baseline608 achieved a mean average precision (mAP) of 82.6% on the Pascal VOC dataset, which is 3.2% higher than the original YOLO v3. More details can be found in Section 4.

**Figure 4:**
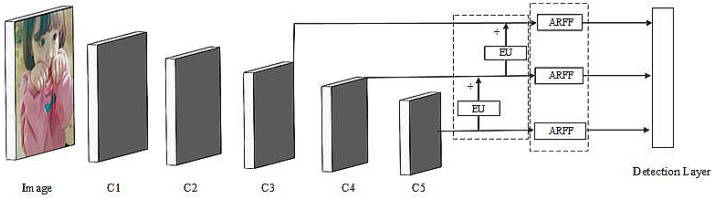
The overall architecture of the ARFF-EU network. C1, C2, C3, C4 and C5 represent the feature map of different RFs, respectively. “+” indicates that the feature map is concatenated in the channel dimension. The dotted box represents the module we embedded.

In this study, we used the modular idea to construct the object detector. As shown in Figure 4, the backbone network was followed by a feature pyramid composed of feature maps of different RFs, which was used to detect objects of different scales. Our implementation maintained the same framework as the baseline and embedded the ARFF modules in the feature pyramid’s three prediction layers to enhance the model’s feature representation ability. The detector in which we embedded the ARFF into the baseline framework is called the ARFF network. In the baseline network, the feature pyramid was constructed through the up-sampling of the feature map. In our implementation, we maintained the same framework as the baseline and used the EU module to replace the up-sampling layer in the feature pyramid to enhance the shallow feature map’s semantic information and improve the detection performance of small objects. The detector in which we embedded the EU into the baseline framework is called the EU network. Finally, we embedded ARFF and EU modules into the baseline framework to build a lightweight, real-time and high-precision object detector, called the ARFF-EU network (ARFF-EU Net). We demonstrate the effectiveness of our proposed model in Section 4.

## 4. Experiments

### 4.1. The Datasets

To verify our proposed model’s effectiveness, we conducted experiments on Pascal VOC and MS COCO datasets, respectively. Pascal VOC is a public benchmark containing 20 labeled object categories. If the IOU of the predicted bounding box and the ground truth bounding box is greater than 0.5, the predicted bounding box is positive; otherwise, it is negative. In this study, we trained the model on the combined Pascal VOC 2007 trainval and Pascal VOC 2012 trainval (VOC 07+12) datasets and evaluated it on the Pascal VOC 2007 test set. MS COCO is a public benchmark containing 80 labeled object categories, which performs more comprehensive calculations by setting various thresholds. Compared with the VOC dataset, the MS COCO dataset contains more small objects, which makes the COCO dataset more challenging. This study uses the COCO train-2017 split (consisting of 115,000 images) for training and COCO val-2017 split (5000 images) for evaluation. We tested our model on a 20,000 image test-dev set. The index used to evaluate the performance of detection was the mean average precision (mAP).

### 4.2. Training Settings

The model was trained and tested on a workstation with four NVIDIA RTX 2080Ti GPUs.

In training, we implemented ARFF Net, EU Net and ARFF-EU Net detectors using the existing Pytorch v1.1 framework with NVIDIA Cuda 10.1 and cuDNN v7.6 toolkits. We followed the training strategies of YOLO v3, including the standard procedures of multi-scale training, mix-up data augmentation, batch normalization, etc. At the same time, we introduced the guided anchor method and “bag of freebies tricks” to build a more powerful baseline network. We used the stochastic gradient descent (SGD) method to minimize the loss function of the original YOLO v3 method to predict the shape of the anchor box and regression of the bounding box. In this study, the input batch size of all models was five, 300 epochs were trained and the mix-up augmentation strategy was turned off [36] in the last 30 epochs. The weight decay was 0.0005, and the momentum was 0.9. We used the MSRA method to initialize all new convolution layers. More details can be seen in the following experiments.

In the test, first, the multi-branch detection layer was used to predict the anchors’ shape. Then, we conducted the classification and bounding box regression following the same pipeline as that in YOLO v3. Finally, non-maximum suppression (NMS) [37] was applied to each class separately, and the threshold of NMS was set to 0.6.

### 4.3. Pascal VOC

We trained the proposed ARFF Net, EU Net and ARFF-EU Net on the Pascal VOC 07+12 dataset in this experiment. We set the model’s input image size to 416 and 608, respectively, and set the initial learning rate to $10^{-4}$. To avoid “nan” in the initial stage of training and make the training process more stable, we used the “warmup” strategy in the first five epochs to gradually increase the learning rate from $10^{-6}$ to $10^{-4}$. In the subsequent training, the cosine learning rate schedule was used to reduce the learning rate from $10^{-4}$ to $10^{-5}$. More detailed settings can be found in Section 4.2.

Table 1 shows the comparison between our results and the state-of-the-art results in the VOC 2007 test set. The baseline is the baseline network used in our study. RFB Net608* was obtained by embedding RFB block into the baseline framework and used the same training platform and strategy as our proposed model.

By embedding the ARFF module into the baseline framework, ARFF416 achieved an mAP of 82.7%, exceeding the performance of SSD513 and DSSD513 using the deep ResNet-101 backbone, and the detection speed of our proposed model was faster. By embedding the EU module into the baseline framework, EU416 achieved an mAP of 82.4%, which was 0.4% higher than Baseline416, and the detection speed was similar (48.0 FPS vs. 48.9 FPS). By embedding ARFF and EU modules into the baseline framework, ARFF-EU@416 achieved an mAP of 83.0%, which exceeded the performance of the advanced two-stage detector CoupleNet using the deep ResNet-101 backbone, and the ARFF-EU@416 was faster. ARFF-EU@416 and RFB Net608* had the same detection accuracy, but the input image size of ARFF-EU@416 was smaller and the detection speed was faster (41.1 FPS vs. 38 FPS).

For larger models, ARFF608 achieved an mAP of 83.3%, which was 0.3% higher than RFB Net608*, and the detection speed of the ARFF608 model was faster (38.5 FPS vs. 38 FPS). EU608 achieved an mAP of 82.9%, representing a performance gain of 0.3% compared to Baseline608. ARFF-EU @608 achieved an mAP of 83.6% at 37.5 FPS, surpassing the state-of-the-art object detectors on the VOC 2007 test set and maintaining real-time speed.

**Figure 5:**
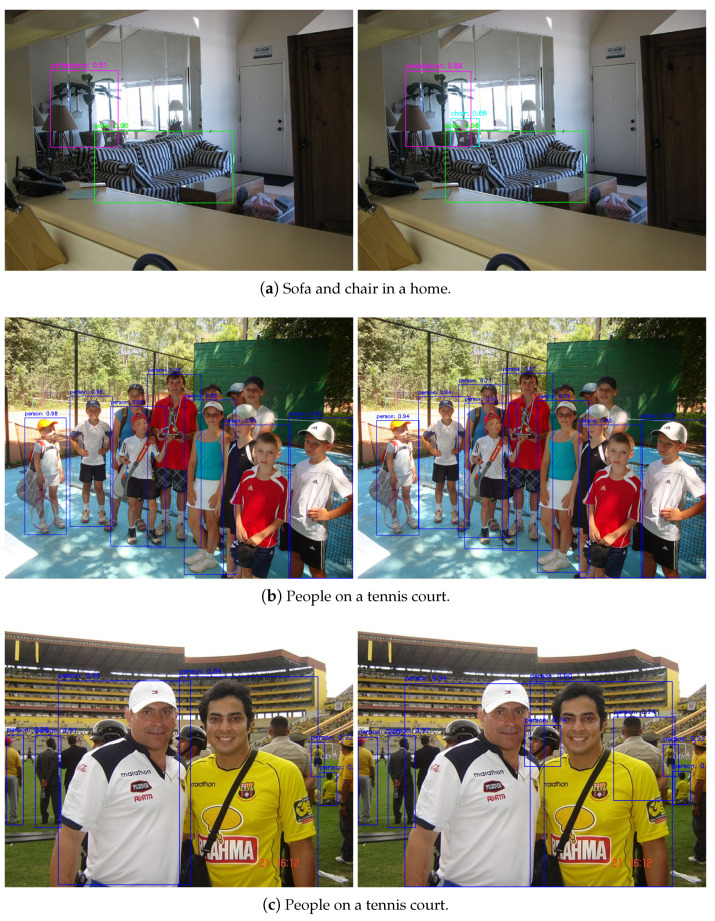
Visualization results of different detectors on the Pascal VOC 2007 test set. On the left is the visualization result of the baseline network, and on the right is the visualization result of the proposed network.

To better understand the ARFF and EU modules, we present some qualitative results in Figure 5. We first present the image using Baseline608 and EU608, as shown in Figure 5a. It can be seen from the figure that the EU608 could detect chairs better than Baseline608, which demonstrates that the EU module improved the model’s detection performance for small objects. Then, we used Baseline608 and ARFF608 to visualize images, as shown in Figure 5b,c. Compared with Baseline608, ARFF608 was better at detecting people with occlusion, demonstrating that ARFF improved the detector’s performance with a complex background by enhancing the model’s feature representation ability.

### 4.4. Ablation Study

#### 4.4.1. Adaptive Receptive Field Fusion

To better understand the effectiveness of ARFF, we investigated the impact of each component on the performance of the detector and compared ARFF with some similar structures. As shown in Table 2, by simply embedding ARFF into the multi-scale object prediction layer of the baseline, we can see that ARFF608 achieved an mAP of 83.3% and a gain of 0.7% (from 82.6% to 83.3%), indicating that ARFF is effective at improving the performance of the detector.

Multi-branch receptive field structure: As described in Section 3.1, we used the multi-branch RF structure to simulate different sizes of RFs in the human visual system and enhance the model’s feature representation ability. As shown in Table 2, when embedding the multi-branch RF structure into the baseline, we can see that the mAP was improved by 0.3% (from 82.6% to 82.9%) without significantly reducing the inference speed, which indicates that the proposed multi-branch RF structure enhanced the feature representation ability of the model. Besides, our proposed multi-branch RF method’s performance gain was similar to that of RFB Net608*, but our model’s inference speed was faster, which shows our proposed model’s efficiency.

Receptive field fusion module: As described in Section 3.1, we used the RF fusion module to filter out redundant feature information in different RF branches and combine useful information to enhance the model’s feature representation ability. As shown in Table 2, when we used the RF fusion module to fuse the feature maps generated by the multi-branch RF structure, the model’s detection accuracy was further improved by 0.4% (from 82.9% to 83.3%), which indicates the efficiency of the RF fusion module. Besides, ARFF608 showed better detection accuracy than RFBNet608* (83.3% vs. 83.0%) and a faster speed (38.5 FPS vs. 38.0 FPS).

Comparison with other spatial receptive fields: We also compared ARFF with RFB, Inception and the deformable CNN. As shown in Table 3, ARFF608 achieved an mAP of 83.3% on the VOC 2007 test set, which was 0.3% higher than RFB Net608* (83.3% vs. 83.0%) and was faster (38.5 FPS vs. 38.0 FPS). Simultaneously, the performance of RFB on the VOC 2007 test set was better than Inception and the deformable CNN, and so the feature representation ability of ARFF was better than RFB, Inception and the Deformable CNN. This indicates that the proposed ARFF has a stronger feature representation ability than other RF modules and improves the detector’s accuracy.

#### 4.4.2. Enhanced Up-Sampling

By simply embedding the EU module into the lateral connection of the baseline, EU608 achieved an mAP of 82.9% on the VOC 2007 test set, which was 0.3% higher than Baseline608 (from 82.6% to 82.9%), and the inference time was comparable (43.8 FPS vs. 44.6 FPS), which indicates the efficiency of EU. We further verified the performance of the EU module on the MS COCO test-dev set, as shown in Table 4; the mAP of EU608 on the MS COCO test-dev set was 26.0%, which was 2.8% higher than Baseline608 (from 23.2% to 26.0%), and the inference time was comparable, which indicates that the EU module enhances the semantic information of the shallow feature map and improves the detection performance of the detector for small objects.

#### 4.4.3. ARFF-EU Object Detector

To better understand the effectiveness of the ARFF and EU modules, we investigated each module’s impact on the detector’s performance. As shown in Table 2, ARFF608 achieved an mAP of 83.3%, representing an increase of 0.7% compared to Baseline608 (from 82.6% to 83.3%). EU608 achieved an mAP of 82.9%, which was an increase of 0.3% compared to Baseline608 (from 82.6% to 83.9%). ARFF-EU @608 achieved an mAP of 83.6%, which was 1% higher than Baseline608, while maintaining real-time speed (from 82.6% to 83.6%). This indicates that ARFF and EU modules are effective for detection.

### 4.5. Microsoft COCO

To further verify ARFF and EU modules’ effectiveness, we conducted experiments on the MS COCO dataset. We set the models’ input image sizes to 416 and 608, respectively, and set the initial learning rate to $10^{-3}$. In order to stabilize the training process, we used a “warmup” strategy in the first five epochs of training, and the learning rate gradually increased from $10^{-6}$ to $10^{-3}$. In the subsequent training, we used the cosine learning rate schedule to reduce the learning rate from $10^{-3}$ to $10^{-5}$. More detailed settings can be found in Section 4.2.

Table 4 shows a comparison between our results and the state-of-the-art results in MS COCO. We can see that the mAP and APs of EU416 were 39.6% and 22.2%, which were 2.4% and 3.5% higher than the corresponding reference scores in Baseline416, and the inference time was similar (40.0 FPS vs. 40.8 FPS). The AP of ARFF416 was 40.3%, which was 6.5% higher than RFB Net512, and the input image size of our model was smaller than RFB Net512. ARFF-EU@416 achieved an AP of 41.2%, which exceeded all advanced one-stage object detectors and achieved real-time speed.

For larger models, the APs of EU608 for small objects was 26.0%, which was better than the detection accuracy of existing one-stage object detectors for small objects and maintained real-time speed. ARFF608 achieved an AP of 41.7%, an improvement of 2.7% compared to Baseline608 (from 39.0% to 41.7%). ARFF-EU @608 improved the baseline network’s detection performance for objects of different scales ($AP_{S}$: 26.2% vs. 23.2%, $AP_{M}$: 44.8% vs. 41.7%, $AP_{L}$: 52.9% vs. 50.8%) and maintained real-time speed. ARFF-EU @608 achieved an AP of 42.5%, which exceeded the advanced FCOS-800 using the deep ResNet-101-FPN backbone and was faster (33.7 FPS vs. 13.5 FPS). Although the detection accuracy of SNIPER is higher than ARFF-EU @608, its detection speed is only 5 FPS, which cannot meet the real-time requirements.

**Figure 6:**
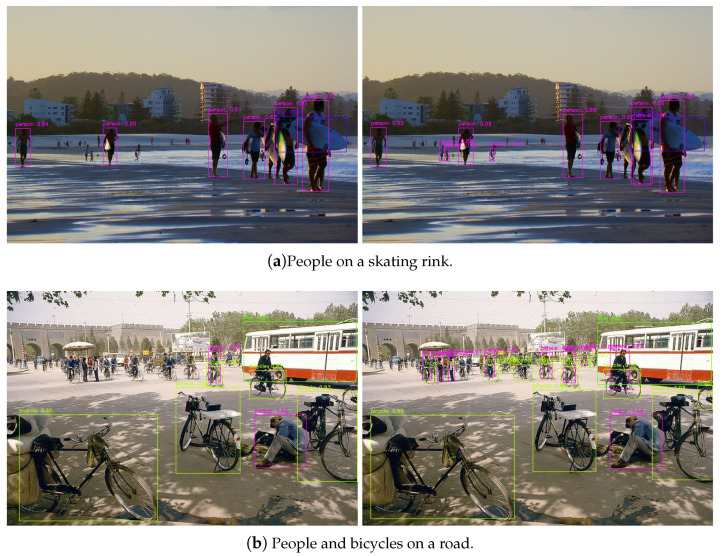
Visualization results of different detectors on the MS COCO test-dev set. On the left is the visualization result of the baseline network, and on the right is the visualization result of the proposed network.

To better understand the ARFF and EU modules, we visualize some images in the MS COCO test-dev use and show the results in Figure 6. We first used Baseline608 and ARFF608 to create the picture. As shown in Figure 6a, compared with Baseline608, ARFF608 was able to detect people with occlusion and smaller objects in the distance better, indicating that the ARFF module enhanced the model’s representation ability and improved the performance of the detector. Then, we used Baseline608 and EU608 to create the picture in Figure 6b. EU608 performed better for bicycles and people with smaller sizes in the distance, which shows that EU608 enhanced the detection performance of small objects.

**Table 4:** Detection performance in terms of AP (%) and FPS on the MS COCO test-dev set.

Method	Backbone	FPS	GPU	*AP*	*AP*_50_	*AP*_75_	*AP_S_*	*AP_M_*	*AP_L_*
*Two-stage Detectors:*									
Faster w FPN	ResNet-101-FPN	11.0	V100	39.8	61.3	43.3	22.9	43.3	52.6
Mask R-CNN	ResNext-101-FPN	6.5	V100	41.4	63.4	45.2	24.5	44.9	51.8
Cascade R-CNN	ResNet-101-FPN	9.6	V100	42.8	62.1	46.3	23.7	45.5	55.2
SNIPER	ResNet-101	5.0	V100	46.1	67.0	52.6	29.6	48.9	58.1
*One-stage Detectors:*									
SSD512	VGG	22	Titan X	28.8	48.5	30.3	-	-	-
RFB Net512	VGG	33	Titan X	33.8	54.2	35.9	16.2	37.1	47.4
CornerNet-511	Hourglass-104	5	Titan X	40.5	56.5	43.1	19.4	42.7	**53.9**
YOLO v3 @416	Darknet-53	45.8	2080Ti	31.0	-	-	-	-	-
Baseline416	Darknet-53	40.8	2080Ti	37.2	58.1	40.2	18.7	40.3	52.6
EU416	Darknet-53	40.0	2080Ti	39.6	59.6	43.0	22.2	42.6	54.7
ARFF416	Darknet-53	37.2	2080Ti	40.3	60.5	44.0	21.5	43.9	56.2
ARFF-EU @416	Darknet-53	36.6	2080Ti	41.2	61.6	44.8	24.1	44.4	50.8
RetinaNet800	ResNet-101-FPN	9.3	V100	39.1	59.1	42.3	21.8	42.7	50.2
FCOS-800	ResNet-101-FPN	13.5	V100	41.0	60.7	44.1	24.0	44.1	51.0
YOLO v3 @608	Darknet-53	40.6	2080Ti	33.0	57.9	34.4	18.3	35.4	41.9
Baseline608	Darknet-53	37.1	2080Ti	39.0	60.4	42.3	23.2	41.7	50.8
EU608	Darknet-53	36.8	2080Ti	41.0	61.5	44.6	**26.0**	43.7	51.2
ARFF608	Darknet-53	34.2	2080Ti	41.7	61.8	45.6	25.4	44.7	52.6
ARFF-EU @608	Darknet-53	**33.7**	2080Ti	**42.5**	**62.4**	**45.7**	**26.2**	**44.8**	52.9

## 5. Discussion

Adaptive receptive field fusion: In this study, we compared the performance of ARFF Net with other advanced detectors. It can be seen from Table 1 that ARFF416 achieved an mAP of 82.7% on Pascal VOC, which was an improvement of 0.7% (from 82.0% to 82.7%) compared to the Baseline416. ARFF608 achieved an mAP of 83.3%, surpassing other advanced detectors’ performance on the Pascal VOC and maintaining real-time speed, which shows that the ARFF module improves the detector’s performance. As shown in Table 2, ARFF608 achieved an mAP of 83.3% on Pascal VOC, which was 0.3% higher than RFB Net608* (83.3% vs. 83.0%) and faster (38.5 FPS vs. 38.0 FPS). This indicates that the ARFF module has a stronger feature representation ability than the RFB block, which enhances the model’s feature representation ability and improves the detector’s performance. At the same time, it can be seen from Table 4 that ARFF416 and ARFF608 achieved AP scores of 40.3% and 41.7% for MS COCO, respectively, which were 3.1% and 2.7% higher than the scores of the corresponding baseline network. This shows that the ARFF module is universally applicable for different data sets, which can enhance the model’s feature extraction ability and improve the detector’s performance.

Enhanced up-sampling: In Table 2 and Table 4, we compare the performance of EU Net with other advanced detectors. As can be seen from Table 2, EU608 achieved an mAP of 82.9% on the Pascal VOC, an improvement of 0.3% (from 82.6% to 82.9%) over Baseline608, while the inference time was basically unchanged. This indicates that the EU module is simple and efficient. Table 4 shows that the AP and APs of EU416 were 39.6% and 22.2%, respectively, which were 2.4% and 3.5% higher than the corresponding reference scores of Baseline416, and the inference time was similar (40.0 FPS and 40.8 FPS). The detection accuracy of EU608 for small objects was 26.0%, which was 2.8% higher than the Baseline608 (from 23.2% to 26.0%) and exceeded the detection accuracy of the existing advanced one-stage object detectors for small objects. This shows that our EU module improves the detection accuracy of small objects by enhancing the semantic information of the shallow feature maps in the feature pyramid without significantly increasing the computational cost.

Comparison with other state-of-the-art detectors: We assembled ARFF and EU modules on top of YOLO v3 to build a real-time, high-precision and lightweight object detection system called ARFF-EU Net. We evaluated ARFF-EU Net on Pascal VOC and MS COCO sets, respectively, and compared it with advanced object detectors in Table 1 and Table 4. As shown in Table 1, ARFF-EU@416 achieved an mAP of 83.0% on Pascal VOC, which exceeded the performance of the advanced two-stage detector CoupleNet using the deep ResNet-101 backbone, and the ARFF-EU@416 was faster. ARFF-EU @608 achieved an mAP of 83.6% at 37.5 FPS, surpassing the state-of-the-art object detectors on the Pascal VOC and maintaining real-time speed. Table 4 shows that ARFF-EU @416 achieved an AP of 41.2% on the MS COCO set, which exceeded the performance of all advanced one-stage object detectors and achieved real-time speed. ARFF-EU @608 achieved an AP of 42.5%, which exceeded the advanced FCOS-800 using the deep ResNet-101-FPN backbone and was faster (33.7 FPS vs. 13.5 FPS). Although the detection accuracy of SNIPER is higher than ARFF-EU @608, its detection speed is only 5 FPS, which cannot meet the real-time requirements. This study shows that the lightweight ARFF-EU Net significantly improves the detector’s performance (from 39.0% to 42.5%) while maintaining real-time speed and achieving the development of advanced, very deep detectors.

## 6. Conclusions

In this study, we propose ARFF and EU modules to improve the model’s feature representation ability and construct an ARFF-EU Net object detector. We evaluated the modules and the detector on Pascal VOC and MS COCO datasets, respectively. The experimental results indicate that the EU module enhances the semantic information of the shallow feature map in the feature pyramid and improves the detector’s performance for small-object detection. The ARFF module enhances the feature representation ability of the RF module and improves the detection performance of the model for objects at different scales. The lightweight ARFF-EU Net we constructed greatly improves the detector’s performance (from 39.0% to 42.5%) while maintaining real-time speed and achieves the performance of an advanced, very deep detector.

## Figures and Tables

**Table 1:** The object detection methods are compared on the Pascal VOC 2007 test set. CNN: convolutional neural network. mAP: mean average precision; EU: enhanced up-sampling; ARFF: adaptive receptive field fusion; RFB: receptive field block.

Method	Backbone	Dataset	FPS	GPU	mAP(%)	Input Size
*Two-stage Detectors:*						
Faster R-CNN	VGG	07+12	7	Titan X	73.2	600×1000
R-FCN	ResNet-101	07+12	12	Titan X	79.5	600×1000
CoupleNet	ResNet-101	07+12	**9.8**	Titan X	**81.7**	600×1000
*One-stage Detectors:*						
SSD512	VGG	07+12	19	Titan X	78.5	512×512
SSD513	ResNet-101	07+12	7.5	Titan X	81.1	513×513
FSSD512	VGG	07+12	35.7	1080Ti	80.9	512×512
DSSD513	ResNet-101	07+12	5.5	Titan X	81.5	513×513
YOLO v3	Darknet-53	07+12	-	2080Ti	79.4	608×608
RFB Net608*	Darknet-53	07+12	38	2080Ti	83.0	608×608
Baseline416	Darknet-53	07+12	48.9	2080Ti	82.0	416×416
EU416	Darknet-53	07+12	48.0	2080Ti	82.4	416×416
ARFF416	Darknet-53	07+12	42.8	2080Ti	82.7	416×416
ARFF+EU @416	Darknet-53	07+12	41.1	2080Ti	83.0	416×416
Baseline608	Darknet-53	07+12	44.6	2080Ti	82.6	608×608
EU608	Darknet-53	07+12	43.8	2080Ti	82.9	608×608
ARFF608	Darknet-53	07+12	38.5	2080Ti	83.3	608×608
ARFF+EU @608	Darknet-53	07+12	**37.5**	2080Ti	**83.6**	608×608

**Table 2:** Test of the effectiveness of various modules on the Pascal VOC 2007 test set.

	Baseline608	RFB Net608*		ARFF608	EU608	ARFF-EU @608
+RFB		🗸				
+Multi-branch RF			🗸	🗸		
+RF Fusion (ARFF)				🗸		🗸
+EU					🗸	🗸
mAP (%)	82.6	83.0	82.9	83.3	82.9	83.6
FPS	44.6	38	42	38.5	43.8	37.5

**Table 3:** Comparison of different receptive field blocks on the VOC 2007 test set.

Architecture	VOC 2007 Test Set mAP (%)
ARFF608	83.3
RFB Net608*	83.0
RFB	80.1
Inception	78.4
Deformable CNN	79.5

## Data Availability

The dataset is available on request from the authors.
